# Evaluation of Reporting Methods for Assessment and Surgical Planning of Perianal Fistulas

**DOI:** 10.1007/s10278-025-01524-4

**Published:** 2025-05-13

**Authors:** Sarra Kharbech, Nabil Sherif Mahmood, Ma’mon Qasem, Julien Abinahed, Amal Alobadli, Mohamed Abunada, Omar Aboumarzouk, Abdulla Al Ansari, Shidin Balakrishnan, Nikhil Navkar, Adham Darweesh

**Affiliations:** 1 Hamad Medical Corporation, Doha, Qatar; 2 Weil Cornell Medicine Qatar, Doha, Qatar; 3 College of Medicine, Qatar University, Doha, Qatar; 4 Glasgow University, Scotland, UK

**Keywords:** Perianal fistula, Surgical planning, Magnetic resonance imaging (MRI), Three-dimensional (3D) visualization

## Abstract

Perianal fistula is a complex condition where surgeons conduct surgeries based on the mentally mapped images they created from the information found in the radiology report. If not properly treated, a fistula could reoccur. To reduce the chances of reoccurrence, a patient-specific, visual, and accurate depiction of the internal tracts in relation to the pelvic floor is required. A three-dimensional (3D) parametric model generation software was previously developed and evaluated successfully with radiologists. In this paper, the software output is evaluated with two colorectal surgeons for 10 fistula cases. The paper compares three reporting different modes: (1) 3D models only, (2) conventional radiology report and picture archiving and communication system (PACS) magnetic resonance (MR) images, and (3) 3D models + standardized radiology report. The percentage of agreement between surgeons across cases and cognitive load are the primary metrics used for evaluation. Mode 3 superseded both modes 1 and 2, meaning that surgeons prefer to see a 3D model along with a standardized report to plan a case’s surgical intervention. Mode 1 superseded mode 2, which also shows surgeons preference to inspect a 3D model rather than inspecting cases the conventional way. Surgeons’ agreement in opinions across cases in mode 3 was 85%, whereas it was 18% and 5% in mode 1 and mode 2, respectively. This shows that information was conveyed more consistently across surgeons in mode 3. NASA TLX tests show that surgeons had the least cognitive load while working with mode 3, followed by mode 1 and then mode 2. Overall, the findings indicate that 3D models, even without radiologists’ written input, outperform the current standard practice of delivering unstructured radiology reports alongside raw PACS images.

## Introduction

Perianal fistula is a condition that manifests as an abnormal connection between the anorectal canal and the perineum skin. It is a condition that affects mainly male adults [1] twice as likely to be affected compared to females [2] with a prevalence of 1 per 10,000 males in their 40s [3]. Perianal fistula causes significant morbidities and requires follow-ups and treatment. The cryptoglandular hypothesis, though never proven, is still considered the best explanation for the etiopathogenesis of perianal fistula formation, where anal gland sepsis and the formation of an anorectal abscess are the initiating event [4]. Obesity, diabetes, and a history of anorectal surgery are some of the reported risk factors [2, 4]. Crohn’s disease is a chronic inflammatory bowel disease, with anal fistula being a common complication and an incidence of 21–23%. A perianal fistula is formed when anal glands get infected, progressing to an abscess, which then does not get adequately treated or drained. That may occur in 40% of the cases [5].

Multiple fistula classifications have been used to categorize fistulas for surgical intervention. Parks’ et al. [6] and St. James University by Morris J et al. [7] classifications are the most common ones. Table 1 shows both classifications.

**Table 1:** Most common fistula classifications and their classification basis

Fistula classifications	
Parks’ et al. [6]	St. James University by Morris J et al. [7]
Based on the tract’s path from the anal mucosa to the perineal skin, with respect to the most outer striated muscle layer [1]	5-grade classification based on the radiological anatomy and its relation to the sphincter mechanism and the presence of secondary tracts and their relation to the levator ani
Intersphincteric	Grade 1: simple linear intersphincteric fistula
Trans-sphincteric	Grade 2: intersphincteric fistula with abscess or secondary tract
Suprasphincteric	Grade 3: trans-sphincteric fistula
Extrasphincteric	Grade 4: trans-sphincteric fistula with abscess or secondary tract within the ischioanal fossa
Submucosal (was recently added)	Grade 5: supralevator and translevator disease

Clinical imaging is used to inspect the fistulous tracts and determine its type to address it with the most suitable case management. Imaging modalities used can be endoanal ultrasound, CT scan, and CT fistulogram or MRI [2]. The latter remains the gold standard for preoperative assessment, as it offers high resolution and multiplanar imaging [8]. Drainage of the abscesses mostly requires a surgery. Failing to address the case properly may cause recurrence and further complications.

Due to the complex nature of the fistulous tracts, visualizing them helps surgeons plan the intervention. We proposed a perianal fistula 3D visualization tool [8] that automatically reconstructs 3D models from MRI images and would allow radiologists to depict simple and complex fistulous tracts for surgeons’ use.

In this manuscript, we use the software’s 3D outputs in some of the reporting modes and compare them with other existing ones. We then present the findings and recommend the most effective solution, specifically the one that best enabled the colorectal surgeons to visualize the fistula case and determine the appropriate intervention.

## Related Work

Understanding the complex fistula anatomy is challenging. After inspecting a subjective radiology report, surgeons need to mentally map the fistulas in relation to the surrounding pelvic structures and plan the procedure accordingly. Inaccurate interventions could result in recurrences, one of the most common perianal fistula complications. Over the years, there have been many attempts to mediate the communications between radiologists and surgeons to minimize complications. Table 2 summarizes MRI-based communication methods articles and categorizes some of them. Categorization is based on how the information is presented to the surgeon: (1) on-screen 3D model, (2) physical 3D-printed reconstruction models, and (3) structured textual report. Table 3 defines each of the communication methods.

**Table 2:** Existing similar studies that used different communication tools to mediate communications between surgeons and radiologists, highlighting the time taken by many methods to take it from MRI to final representation

Refs	Communication method	Imaging source	Evaluated metrics	Time taken to generate final representation	Key findings
[8]	Onscreen 3D models	MRI	Time to interpret different 3D representations	Standard 3D approach: 105 mins (~ 1 hr and 45 mins)	Parametric models take less time to produce than standard 3D approach. See Fig. 1 for difference
				Parametric model generation: 25 mins	
[9]	Onscreen 3D models	MRI, endoanal US	Intraoperative visual assistance	NA	3D models helped surgeons in the preoperative planning and during surgery. 3D models showed excellent anatomical correlation with intraoperative findings
[10]	Onscreen 3D models	MRI	Accuracy of readings by radiologists and surgeons for various 3D anatomical features	20 to 40 mins depending on case complexity	The results show high diagnostic inter-observer agreement between radiologists and surgeons, with 4 out of 5 readings with more than 92% agreement The highest agreement was while reading supra-levator extension with 100% agreement, and the least was while reading side branches with 88% agreement
[11]	Onscreen 3D models	MRI	Subjective feedback: How helpful were seeing and interacting with 3D models on the phone	NA	A satisfactory low-cost solution. Surgeons can manipulate 3D reconstructions on their smartphone pre and intraoperatively. It was helpful during cannulation of the fistula tracts/internal orifice and for the identification of neighboring structures. No signs of local fistula recurrence or abscesses after 8 months follow-up
[12] [13] [14]	Physical 3D-printed reconstruction models	MRI	Subjective feedback: How useful are the 3D printed models?	85 mins (~ 1 hr and 25 mins)	There were similar benefits to printed 3D models and 3D images. Junior surgeons benefit from it the most in terms of education and preoperative planning. Models also facilitated the communications with patients, with over 75% of them asking to keep the model
[15]	Physical 3D-printed reconstruction models	MRI	Cohen’s kappa statistic to evaluate (1) consistency between the radiologist and the surgeons and (2) agreement between the two surgeons in terms of classification, complexity, and location of the internal orifice Interclass correlation coefficients (for continuous values) evaluate percentage of involved external anal sphincter in transsphincteric fistulas	325 mins (~ 5 hrs and 25 mins)	Authors segment the MRIs, generate the 3D physical representation of the case, and then re-scan the printed 3D model to validate if they are a true representation of the MRIs
[16]	Structured textual reporting	MRI	No evaluation reported	NA	A uniform standard report for objective and informative reporting. It covers 8 key fistula descriptors. A study is undergoing with study number NCT04541238 to assess the effectiveness, reproducibility, and acceptability of the new reporting template
[17]	Structured textual reporting	MRI	No evaluation reported	NA	A systematic radiology report that offers a synoptic view for the case. It covers 11 key fistula descriptors
[18]	Structured textual reporting	MRI	Compare the clarity and usefulness of the conventional report compared to the structured one	NA	The structured report was significantly better than the conventional report. It presented clearer information and was better evaluated

**Table 3:** Communication methods and their definitions

A communication method = a way to mediate the communications between radiologists and surgeons		
No	Communication method	Definition
1	On-screen 3D models	An objective method to visualize and manipulate the fistula’s complex anatomy. It is presented in relation to the surrounding anatomical structures
2	Physical 3D-printed reconstruction models	An objective method where a 3D representation of the fistula case is printed and presented to surgeons and patients
3	Structured textual report	Subjective method where the radiologist fills in a report based on the understanding from PACS images

From the above surveyed articles in Table 2, 3D is the most used method to visualize fistula anatomy with all its variations (3D on screen, 3D printed, etc.). This is evident in a survey by [14] stating that 85% of surveyed international surgeons think that 3D representation would be useful for perianal fistula surgery, and 88% would use the feature if available. During consultations, 96% of the patients appreciated them and want to see them in future ones [19]. The table also shows different 3D solutions with different turnaround times (i.e., time taken to generate 3D representation from MRIs). Our solution [8] showed one of the least times taken to generate the 3D model.

**Fig. 1:**
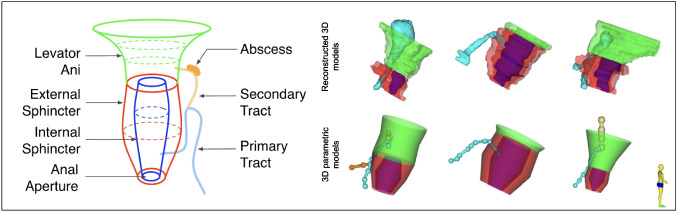
**a** A typical representation of a 3D parametric model. **b** The difference between the reconstructed 3D models and the 3D parametric models for three cases

Although a picture is worth a thousand words, written clinical reports are an irreplaceable reference to thoroughly understand the fistula anatomy, its classification, prognosis, etc. Proper reporting is crucial to decide on the proper case management and track treatment response [16]. Traditional narrative reports are prone to being incomplete and being understood differently by different readers. It is also often associated with high inconsistency in the language, length, and style [20] due to the lack of standardization, which makes it harder to extract or emphasize key clinical findings. Structured and synoptic reporting is being overwhelmingly preferred by radiologists [21] as they ensure reports completeness, comparability, minimize ambiguity, standardize the report structure and terminologies, and facilitate automated functions [22] such as data extractions for research purposes. A study shows that narrative reports significantly miss key features as compared to structured ones [23], which further justifies the need to use structured reports, replacing the narrative one.

In this study, we propose to test the utility of providing the surgeons with a structured report along with 3D images as compared to the standard of conventional MRI images with a text-based report or 3D models alone. To the best of our knowledge, such a combination has never been tested nor evaluated, which makes this work novel.

### Current Practice in Hamad Medical Corporation

The current practice in Hamad Medical Corporation’s (HMC) radiology department is similar to most hospitals worldwide. A radiologist inspects the PACS images and writes a report accordingly. The surgeon then reads the report and often uses the PACS images and tries to mentally map the tracts in relation to the pelvic structures and plan the surgery accordingly.

## Materials and Methods

The research comprises three integral components. The first component involves the design and development of the software. The second component involves the acquisition of input from radiologists. The third one is the evaluation conducted by colorectal surgeons. In this manuscript, the focus is on the third component. Our previous work involved the development of a software that generates parametric models, which look like 3D models. We aim to evaluate it against other available approaches. We chose two more modes (modes are explained further in the below sections) as comparators: one mode is the current HMC’s practice. Another one is combining the 3D parametric model with the structured textual report published in this manuscript [16]. The report was adapted by discarding the visual depictions and using only the synoptic reporting part for fair comparison reasons. A template of the report is shown in Table 4. The report has eight key fistula-related descriptors, such as primary and secondary tracts, residual abscess, and sphincter morphology. Details on each of the descriptors are available in [16].

**Table 4:**
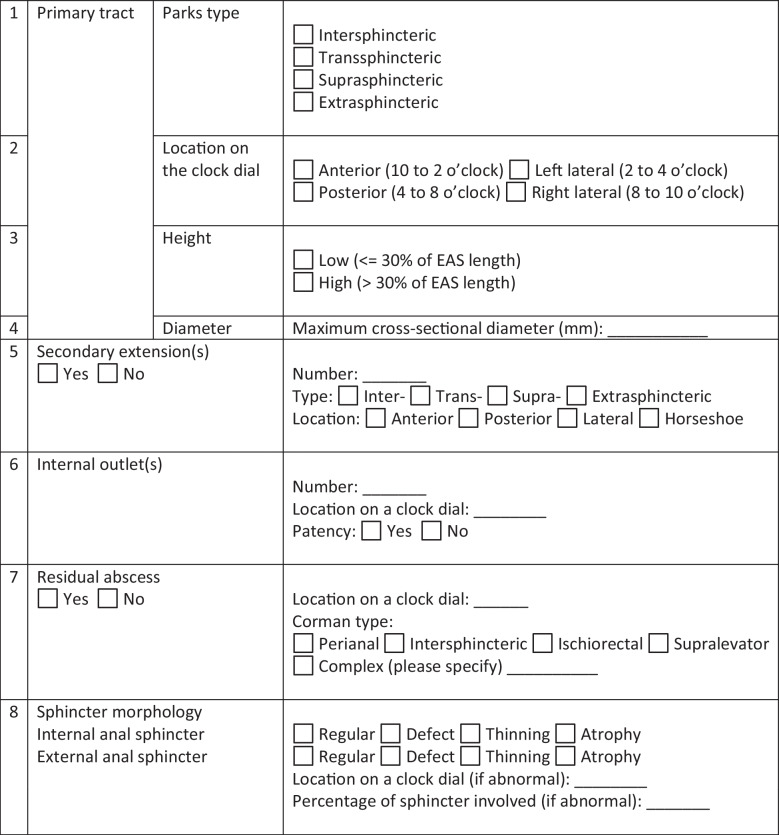
The standardized report used in mode 3. More details on each of the descriptors are available in the original paper

### Software

**Fig. 2:**
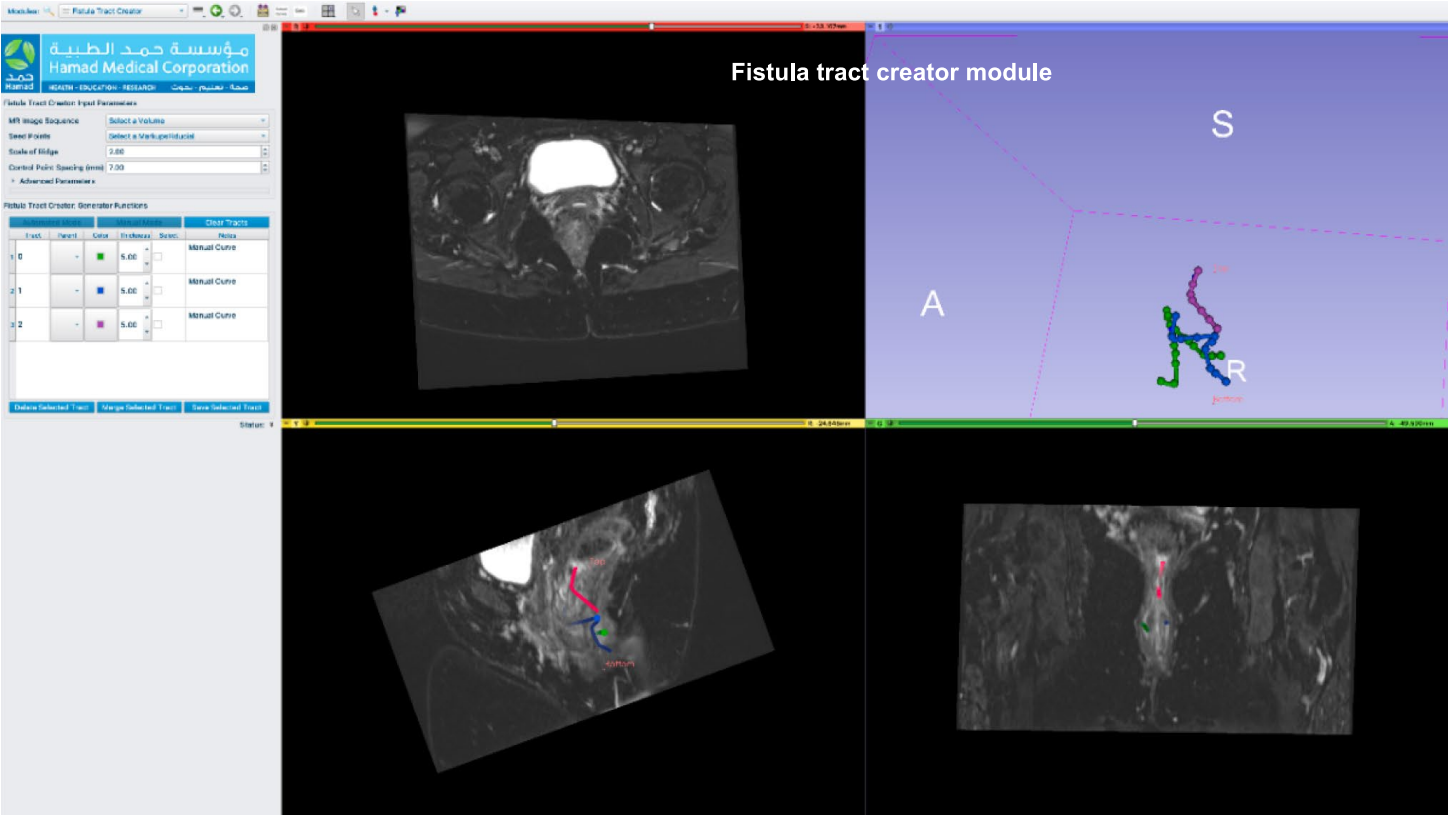
A screenshot from Fistula software showing the fistula tract creator module

**Fig. 3:**
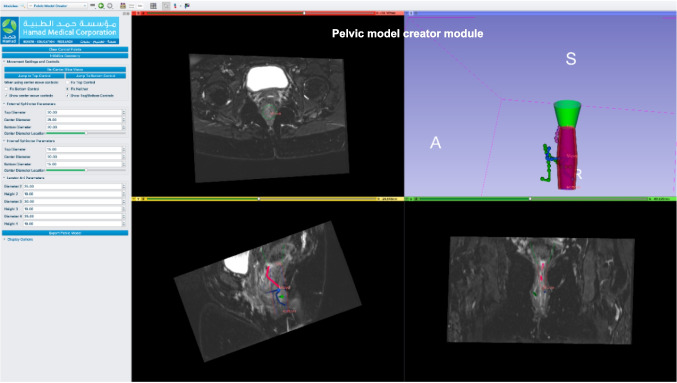
A screenshot from Fistula software showing the pelvic model creator module

A software named “HMC Fistula” was developed on top of a software called “Slicer 3D [24]”: a free, open-source medical/biomedical software by Kitware [25] that is designated to segment, visualize, process, register, and analyze medical, biomedical, and 3D images. In addition to its native modules, two new modules were developed and added specifically to serve our needs. The first module was “Fistula Tract Creator.” This entailed marking fiducial points on the tracts visible in coronal, sagittal, and axial MR images. Subsequently, tracts were generated, allowing radiologists to further annotate details such as primary or secondary tracts and abscesses. A notes section was provided for additional comments or annotations. After saving the tracts, radiologists proceeded to the “Pelvic Model Creator” module to construct a patient-specific pelvic model. Control points were placed on MR images to delineate the external sphincter, internal sphincter, and levator ani. The external sphincter parameters can be changed using four different control points: top diameter, middle diameter, bottom diameter, and center diameter location. Similarly, for the internal sphincter, the parameters can be adjusted using four control points: top diameter, middle diameter, bottom diameter, and center diameter location. The levator ani was split over three adjustable parts. Each part has a controllable height and diameter. Upon initiating geometry, a parametric 3D pelvic model was instantly generated. Figures 2 and 3 show screenshots from the software.

### Modes

**Fig. 4:**
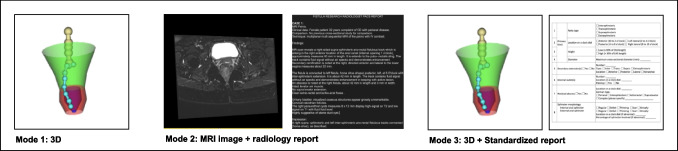
Three reporting modes used in the study. Mode (1) 3D, mode (2) MRI image + radiology report, and mode (3) 3D + standardized report

This paper compares three distinct reporting modes. The modes are summarized in Fig. 4 and described below:

- Mode 1 presents the surgeon with a 3D depiction of fistulous tracts and a 3D pelvic floor to highlight their spatial relationship. No textual report is provided in this mode.
- Mode 2 adopts the conventional reporting method: the examination of PACS images alongside the textual radiology report.
- Mode 3 involves the surgeon reviewing a 3D parametric model of fistulous tracts and a 3D pelvic floor anatomy, complemented by a standardized reporting method (Table 4) for fistula cases adopted from [16].

### Study Design

**Fig. 5:**
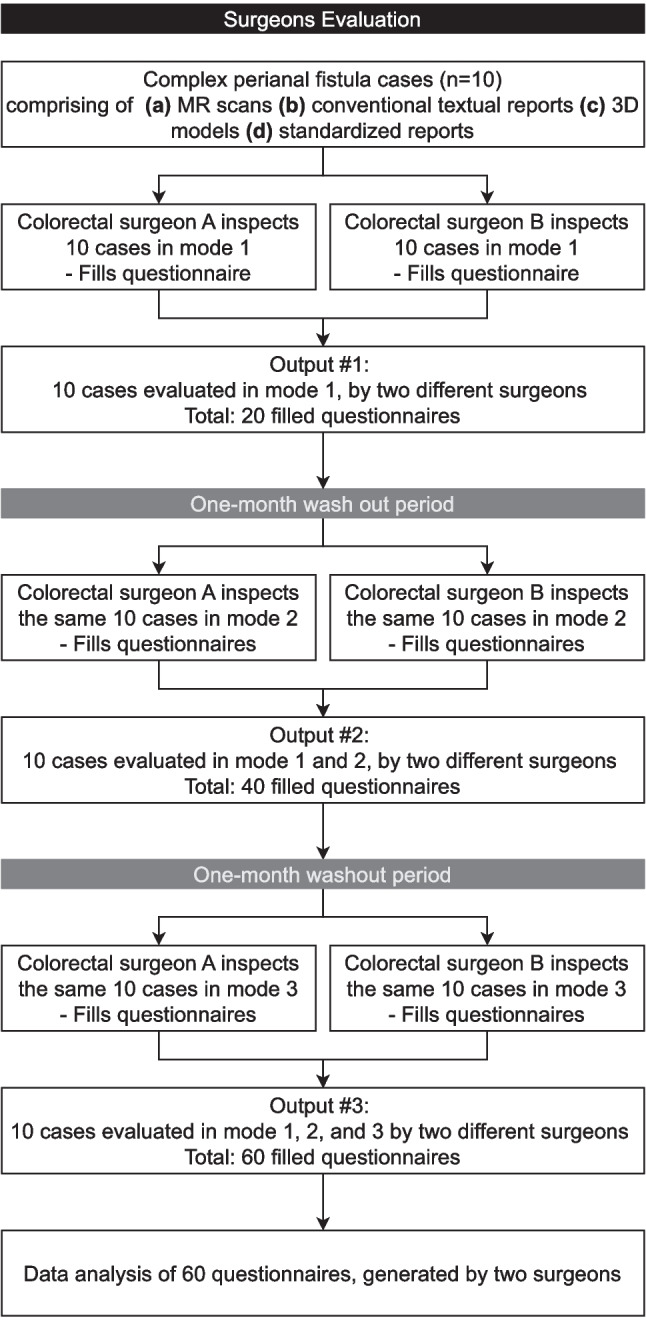
Experiments flow with outputs after each step

In this paper, surgeons’ evaluation results are reported. Figure 5 presents the study design. Surgeons were presented with 10 cases, but one mode at a time, and were requested to work on them individually. In the 1st phase, surgeons inspected all cases by solely viewing a 3D representation of the fistula and filled out questionnaires accordingly. Surgeons had a 1-month washout period, followed by the 2nd phase, where they inspected the same 10 cases by viewing the PACS images and reading the radiology report. After a second 1-month washout period, surgeons performed the 3rd phase, which involved inspecting the 3D representations and reading the accompanying fistula standardized report.

### Surgeons’ Evaluation

Two colorectal surgeons, both with 20 years of experience, were recruited to evaluate cases across three different modes. Ten distinct cases are shuffled and presented to the two colorectal surgeons. The 10 cases were chosen based on their highest similarity measure, particularly the average distance (AD) between two primary tracts’ splines [8]. Evaluations were conducted independently to refrain the surgeons from case discussions. Each one was provided with a dedicated laptop to perform the study.

**Fig. 6:**
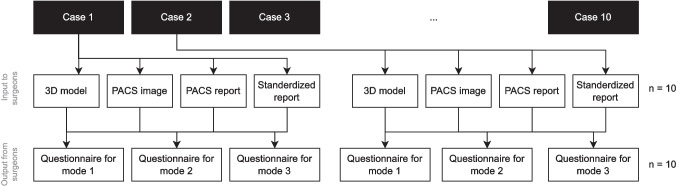
The input and output to surgeons for all fistula cases

As Fig. 6 depicts, one case folder comprises of (a) a 3D illustration of fistula tracts and pelvic model, (b) a distinct patient’s MRI scans, (c) a PACS textual report, and (d) a standardized report. Simultaneously, each surgeon was instructed to complete the case questionnaire for all 10 cases in one mode before transitioning to the next and to take a 1-month break after completing each mode to minimize bias associated with recalling case details and intervention suggestions.

For each case, the surgeons answered questions related to case details and proposed suitable interventions. Surgeons were involved in the design of the questionnaire to ensure the inclusion of clinically relevant questions in it, as well as ensuring that the structured reporting template provided by the radiologists would reflect the same. It is important to get the surgeons on board during the early design phases to ensure the solution caters to their needs.

At the end of each case questionnaire, the NASA Task Load Index (or TLX) questionnaire was appended to the end of each case questionnaire to measure the cognitive load while performing cases in different modes. This questionnaire is a standard and subjective mental workload assessment published by NASA to measure mental workload while a participant is performing a task [26].

All questionnaires were then collected, and the answers were encoded in binary format for further data analysis.

## Results and Discussions

### Data Analysis

After getting all questionnaires from surgeons, data was recorded on Excel and encoded in a binary format: “Yes” is encoded as 1, and “No” is encoded as 0. Table 5 shows a snapshot of the encoded data for the questionnaire.

**Table 5:** A snapshot of the data encoded post questionnaire collection. The complete dataset has 60 rows: 2 surgeons × 3 modes × 10 cases

Surgeon	Case	Mode	Q1	Q2	Q3	Q4	Q5	Q6	Q7	Q8	Q9	Q10	Q11	Q12	Q13
Surgeon 1	Case 1	Mode 1	1	1	1	1	0	0	1	1	1	1	1	0	0
Surgeon 1	Case 1	Mode 2	1	0	1	0	0	0	1	1	1	1	1	1	1
Surgeon 1	Case 1	Mode 3	1	1	1	1	0	0	1	1	1	1	1	1	1
Surgeon 2	Case 1	Mode 1	1	0	1	0	0	0	1	0	0	1	0	0	0
Surgeon 2	Case 1	Mode 2	0	0	0	0	0	0	0	0	0	0	0	0	0
Surgeon 2	Case 1	Mode 3	1	1	1	1	1	1	1	0	1	1	1	1	1

One of the comparison metrics is to calculate the agreement among surgeons for all cases per mode. If two surgeons agree on one answer (i.e., both answers “Yes” or both answer “No”), this is considered an agreement. To automate this, we performed the logical NOT XOR operation for every pair of answers from both surgeons for the same case and the same mode. Table 6 shows all possible inputs and outputs combinations using the logical operation NOT XOR.

**Table 6:** All possible surgeons’ answers combinations using the NOT XOR logical operation

S1 answer	S2 answer	XOR	NOT (XOR)
0	0	0	1 (True)
0	1	1	0 (False)
1	0	1	0 (False)
1	1	0	1 (True)

The sum of “True” is then recorded per mode per question, and agreement % is calculated as follows:

$$\mathrm{Agreement}\%=({~}^{\text{Number of "True" per mode per question }}\!\left/ \!{~}_{\text{Number of cases}}\right.)\times100$$

### Results

Results are displayed in four different graphs. They suggest that mode 3 was the best mode that helped them visualize a fistula case and decide on the most suitable intervention.

**Fig. 7:**
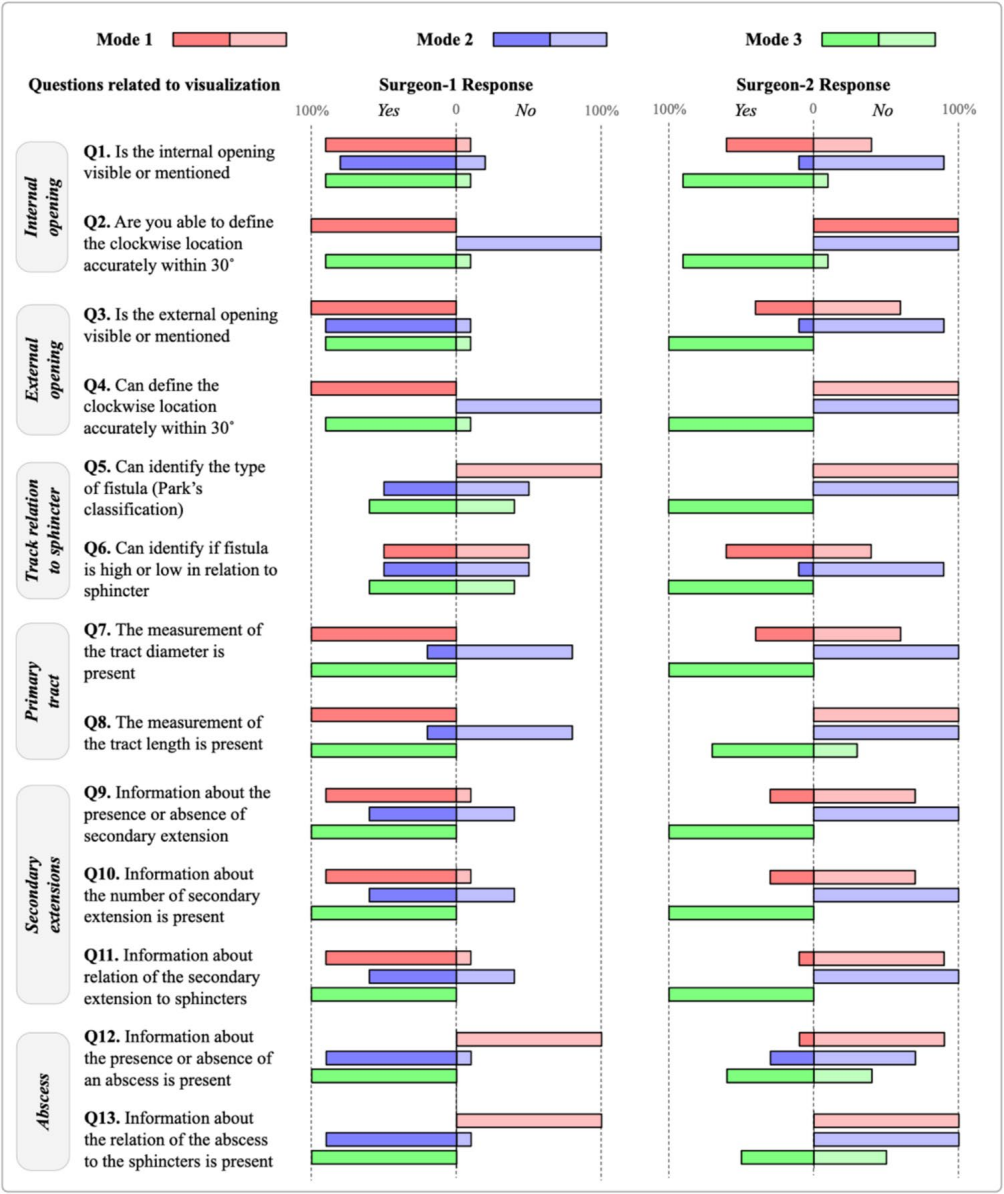
Answers’ summary: the percentage of “Yes” and “No” answers by each surgeon for each question for all 10 cases, categorized per mode

Figure 7 depicts the surgeons’ answers’ summary: the percentage of “Yes” and “No” answers by each surgeon for each question for all 10 cases, categorized per mode. In the figure, it is notably visible that mode 3 has similar responses by both surgeons, which means that this mode led to more consensus in opinions among surgeons. In contrast, modes 1 and 2 had many discrepancies in surgeons’ answers.

**Fig. 8:**
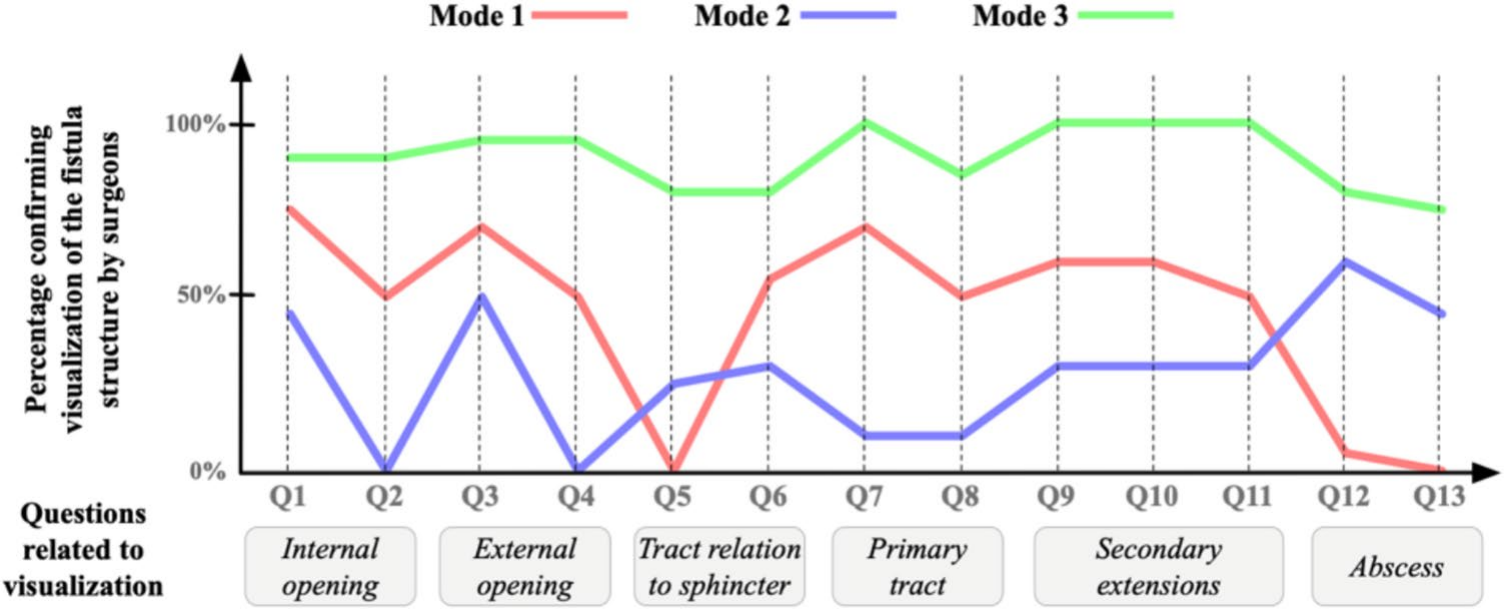
A line graph where the *x*-axis is for the questions and the *y*-axis is the percentage of which surgeons are confirming the visualization of a fistula structure

Figure 8 averages the data shown in Fig. 7 and presents them in a different format. It shows aggregated percentages by which the surgeons confirmed that a particular fistula structure is shown or not. For instance, in question 7 mode 3, both surgeons saw that information about the tract’s diameter was showing. Thus, the green graph point is at 100%. On the other hand, in question 2 mode 1, one surgeon saw that she was able to define the internal opening’s clockwise location accurately within a 30° angle, whereas the other surgeon did not. Thus, the red graph point is at 50%.

As the graph suggests, mode 3 is the only mode where all key fistula structures are mostly visible to all surgeons. Whereas in mode 2, some structures are poorly or not visible for both surgeons, such as the fistula’s Parks classifications. The external opening’s clockwise accurate within 30° angle location is also not visible in mode 2. Comparing the latter modes with mode 3, this mode is more reliable, as all surgeons agree that structures are present with high percentages that drop to 70% at minimum (question 13) but maintain a high percentage for the rest of the fistula structures, reaching 100% in 3 questions (questions 7, 9, and 11).

Through the figure, for modes 1 and 2, the visibility of fistula structures fluctuates significantly and reaches 70% of visualization confirmation maximum. This means that they may not be a reliable source for surgeons to inspect for informative decision-making. Thus, based on surgeons’ evaluations, mode 3 is the best mode that visualizes key fistula structures.

**Fig. 9:**
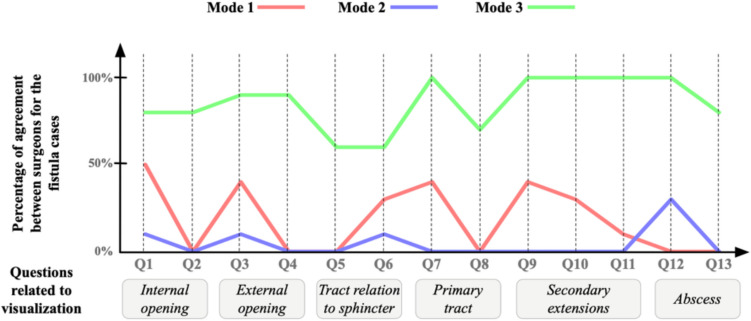
A graph that shows the percentage of agreement between surgeons across cases. The *x*-axis is the questions, and the *y*-axis is the agreement percentage

Figure 9 shows the percentage of positive agreement among surgeons regarding the examined fistula cases. For mode 3, there is a relatively high agreement among surgeons about the cases. The agreement % drops to a minimum of 60% in the questions related to tract relation to sphincter. This suggests that, although their agreement is fair, there should be a way to improve the visualization of this structure. There is also a 100% agreement in questions related to secondary extensions, which means mode 3 visualizes them greatly. For modes 1 and 2, their agreement fluctuates and does not reach above 50% in all the cases. As the graph suggests, the two surgeons have low agreements for all the questions across all cases. This means that the same information presented to surgeons could be interpreted differently by different surgeons, which consequently means that the data is presented inadequately to surgeons for them to make similar decisions. Accordingly, mode 3 is the best mode where all surgeons have very similar opinions consistently.

**Fig. 10:**
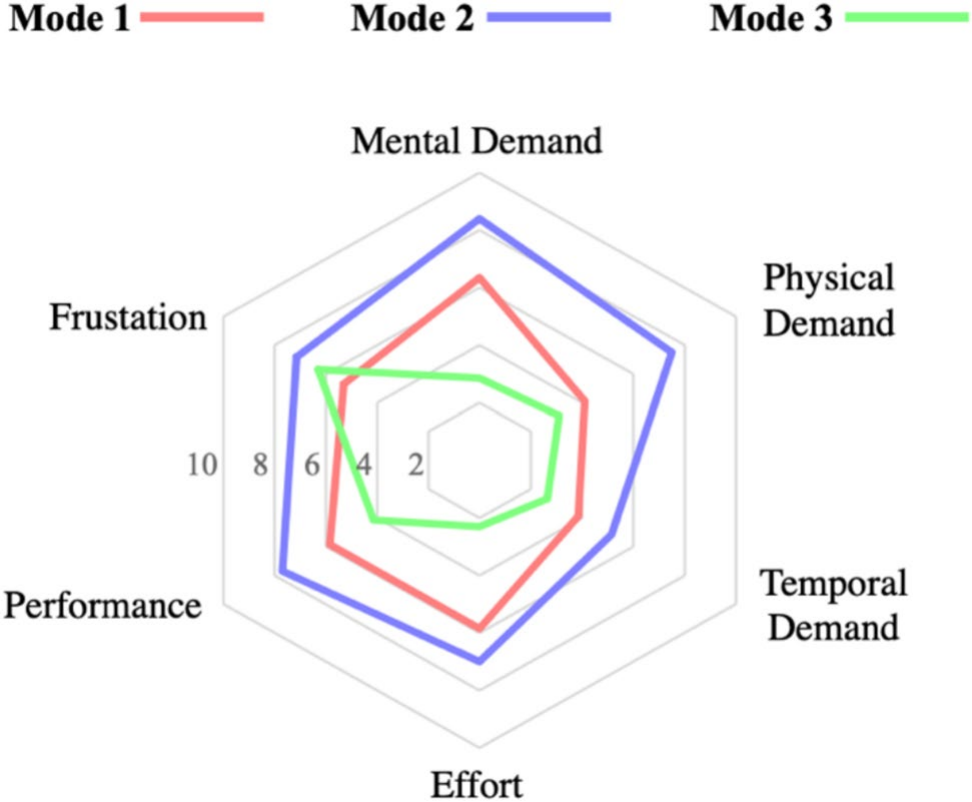
NASA TLX-based cognitive load questionnaire results summary for each mode

Figure 10 shows the results from NASA TLX questionnaire that was used to measure the cognitive load when inspecting the fistula in different modes. The figure summarizes the feedback from both surgeons. To interpret the figure, lines closer to the chart’s center denote low cognitive load, while lines away from the chart’s center denote high cognitive load. As depicted, mode 1 and 2 showed higher cognitive load in all aspects, as compared to mode 3 except in frustration. This is because surgeons were frustrated while dealing with the software, it lags, and they often need to reload the case to view it. This has been noted as a software limitation to be addressed in the next release. It is remarkable that mode 3 is significantly better in lowering the efforts done by surgeons to accomplish the task and better in lowering the mental demand on them to interpret a case. This suggest that surgeons would feel less stressed during case inspection and more confident about choosing the most suitable surgical intervention.

### Discussions

To the best of our knowledge, based on an initial review of the existing literature (Table 3), the proposed combination of 3D visualization coupled with a structured report has not been previously introduced nor systematically evaluated. This work represents a novel contribution towards optimizing communication between radiologists and surgeons by identifying reporting methods that require minimal interpretive effort, reduce bias, and deliver information in a clear, structured, and clinically useful format. Building upon this conceptual foundation, we empirically compared different reporting modes to evaluate how these enhancements translate into practical benefits for surgical interpretation.

Mode 3 superseded modes 1 and 2. This is because surgeons inspect two inputs: (1) they see an interactive patient-specific 3D visualization of the case in addition to (2) a radiology report that had succinct and structured information, rather than a bulk of text. Results show that mode 3 imposed the least cognitive load on surgeons while inspecting cases, as opposed to modes 1 and 2. Although mode 2 had a visual component (i.e., the PACS images), modes 1 and 3 superseded mode 2. This shows that 3D representations are preferred by surgeons compared to traditional imaging, regardless of them being accompanied by a textual report.

Statistical analysis shows that surgeons’ agreements in mode 1 and mode 3 superseded mode 2 in 53.8% and 100% of the cases, respectively. This means that a case’s 3D representation is favorable as it provides information in a way that leads both surgeons to see and interpret information the same way. We can also conclude that the 3D model on its own performed better than the conventional method of reading non-structured radiology reports with PACS images, which is the most used mode in day-to-day practice. There was a better degree of agreement between the surgeons and lesser cognitive load with 3D models alone, as compared to the conventional practice of having non-structured reports with PACS images (Figs. 9 and 10). Even in the absence of textual input from a radiologist, 3D models on their own have a better performance than the current practice. In mode 3, we went a step further and added standardized reports with the 3D models, which outperformed the two other modes. We agree this could either mean standardized reports on their own (which we did not separately test), or coupled with 3D models, would work better than the conventional system.

## Limitations

The study uncovered few limitations. Literature-wise, it is apparent that among all manuscripts reviewed, there is no unified way to evaluate the effectiveness of different communication methods. This made it hard to objectively compare different methods. From Table 2, it is also apparent that there are various efforts towards creating structured textual fistula reports. However, there is still no consensus on what a “full” fistula report is.

The study was also limited by a small sample size, which may affect the generalizability of the findings. Additionally, the evaluation process was conducted by only two surgeons, potentially introducing bias and limiting the diversity of perspectives in assessing the outcomes. A larger sample and a broader panel of evaluators could provide more robust and widely applicable results, which could be done in the future.

When it comes to our developed software, surgeons were not able to easily visually distinguish abscesses from fistula tracts for all cases. In some cases, abscesses were easier to detect than in others. In addition, the software responsiveness was another reported limitation by surgeons. It crashed numerous times, which hindered the surgeons’ effectiveness and increased their frustration to operate, which was apparent in Fig. 10. These limitations are essential to address before the next release.

## Future Work

Further research is needed to determine the optimal combination of reporting methods that best supports surgeons in planning surgical interventions. Future studies should evaluate and compare various modalities, including PACS free-text reports, PACS MR images, structured reports, 3D on-screen representations, and 3D printed models, to identify the combination that minimizes bias while providing the most accurate and reliable information for surgical planning. The study should include more surgeons and more cases to reach better and more accurate conclusions.

We believe our study, though having its weaknesses, does, however, open avenues for further work in this field, such as developing artificial intelligence tools to automatically generate 3D models of fistulous tracts and abscesses with standardized reports, which we know will perform better at the user end, as compared to the current method of providing PACS images with non-standardized free-text reports. We also believe this automated software could be used not only for perianal fistulas, but it could also be tweaked slightly to reconstruct similar complex anatomies, such as the brachial plexus, for better visualization and thus better surgical intervention. Additionally, the reconstructed models could be good material for teaching and training, as they are interactive and a rich source of information where learners are able to manipulate and analyze the generated models.

## Conclusion

Complex perianal fistulae are usually associated with complex anatomical structures. Without adequate visualization and surgical treatment, patients are prone to complications. 3D visualization, coupled with a standardized report, helped surgeons to better mentally map different cases and, thus, make better informed surgical decisions. Throughout the years, researchers have been looking for the most effective way to communicate most of the information MRIs have to offer. The novelty of this work lies in developing and evaluating a combination of communication tools, which are 3D models and standardized reports. The proposed software could be used in many other surgical specialties, especially when complex anatomy is involved. Finally, the generated 3D models are patient-specific, which helps surgeons to better understand the individual anatomy and pathology variations. This also aids them in planning surgeries better and ultimately improves patient outcomes and advances the standards of personalized care.

## Abbreviations

PACS: Picture archiving and communication system
MR/MRI: Magnetic resonance/magnetic resonance imaging
CT: Computed tomography
3D: Three-dimensional
